# Wireless radiation and health: making the case for proteomics research of individual sensitivity

**DOI:** 10.3389/fpubh.2024.1543818

**Published:** 2025-01-10

**Authors:** Dariusz Leszczynski

**Affiliations:** University of Helsinki, Helsinki, Finland

**Keywords:** electromagnetic hypersensitivity, biomarkers, environmental sensitivity, radiation sensitivity, individual sensitivity, provocation studies, proteomics, omics technologies

## Introduction

The current deployment of the fifth generation of wireless communication technology (5G) has reignited the long-standing debate around the possibility of health effects from the radiation emitted by the existing wireless communication devices and networks and the new ones introduced by the 5G. The opposition of the part of society toward wireless communication technologies is caused by the uncertainty of whether this radiation affects humans as well as fauna and flora.

Some of the population considers themselves sensitive to wireless radiation, the so-called electromagnetic hypersensitivity (EHS). Currently, the existence of EHS has not yet been proven scientifically. However, according to the definition of health of the World Health Organization (1) where “*health is a state of complete physical, mental, and social wellbeing and not merely the absence of disease and infirmity*”, any person believing his/her health is affected or sensitive to wireless radiation experiences a health effect caused by the wireless radiation. According to the WHO definition of health, just a belief in having EHS and experiencing non-specific symptoms, physiological and/or psychological, is experiencing the health effects of wireless technology. Hence, it is correct to claim that wireless radiation causes health effects.

The existence of individual sensitivity to wireless radiation has not been proven yet, or disproven because to date performed research is inadequate. Of course, it is challenging to prove things with science as an infinite number of potential confounders and covariates need to be considered. However, science should be used to evaluate the balance of probability whether a hypothesis is plausible. Hence, providing an absolute proof might be elusive. Most of the research on EHS was conducted using psychology methods, asking a person, who is concerned that wireless exposure might affect health of the wireless radiation exposure, how the person feels during the real or the sham exposure. Puzzlingly, the frequent observation that the self-declared EHS person can't feel the wireless radiation and can't recognize when the wireless transmitter emits radiation and when it is not transmitting, is considered ultimate proof that the form of individual sensitivity to wireless radiation called EHS is not caused by wireless radiation exposures. This is questionable as no person, sensitive or not, could feel the ionizing radiation or other non-ionizing radiation like ultraviolet in their environment. Thus, research on EHS and individual sensitivity to wireless radiation, in general, has generated scientifically subjective data, unreliable for public health recommendations or radiation safety limits. On the contrary, logically and *per analogiam* with other environmental factors, individual sensitivity to wireless radiation, which includes EHS, exists as indicated below, and should be studied using biochemical methods.

## Environmental sensitivity

It is well established that different individuals can be differently affected by the same agent of chemical or physical nature. This phenomenon of individual sensitivity can vary greatly among different people due to genetic, physiological, and psychological differences. Individual sensitivity is a well-documented phenomenon in the fields of environmental health, toxicology, and epidemiology.

The broad theory of environmental sensitivity (2) proposes that although all people are sensitive to their environment, some individuals tend to be more sensitive than others. The theory of environmental sensitivity suggests that people vary in their sensitivity to the environment due to differences in their ability to perceive and process information coming from the environment. The more sensitive individuals have heightened perception and deeper processing of external and internal environment stimuli due to genetically influenced physiological and neurobiological differences in the functioning of the internal organs and the central nervous system. One also needs to consider factors such as certain drugs, vaccines, prior exposures to radiation, pesticides, mold, viruses, bacteria and chemicals can sensitize people (3).

Understanding the phenomenon of individual sensitivity to environmental factors, chemicals, and radiation requires a multi-faceted approach that encompasses genetic, epigenetic, immunological, and developmental perspectives. Each individual may respond differently based on these factors, leading to varying health outcomes or lack of these. Individual sensitivity to environmental factors is regulated by:

- Variability in genetic makeup where genetic polymorphisms can affect how individuals metabolize and respond to chemicals and environmental factors. For example, variations in genes responsible for detoxification, such as glutathione S-transferase (GST) genes, can influence susceptibility to cancer from environmental toxins (4).
- Epigenetic factors where epigenetic modifications, which can be influenced by environmental factors, may cause individuals to react differently to exposures. For instance, prenatal exposure to certain environmental toxins has been linked to changes in DNA methylation patterns that impact health outcomes (5).
- Age and developmental factors where younger populations, particularly infants and children, often show higher sensitivity to environmental toxins. Developmental stages can influence how the body processes and responds to these chemicals (6).

Here are a few examples of health effects regulated by the variations in individual sensitivity to the environment:

- Immune system variability where individual differences in immune system function can lead to varying responses to environmental chemicals and radiation. For example, some individuals may exhibit allergic reactions or more severe effects from pollutants, which can be due to genetic predisposition or previous exposure history (7).
- Allergies and asthma where some individuals are highly sensitive to environmental allergens such as pollen, dust mites, mold, and pet dander, leading to allergic reactions or asthma. Genetic predisposition plays a significant role in this sensitivity (8).
- Chemical sensitivity, where some individuals experience multiple chemical sensitivity (MCS), where exposure to low levels of various chemicals in the environment leads to symptoms affecting multiple organ systems. This sensitivity can vary widely among individuals (9).
- Environmental stressors and mental health, where factors such as noise pollution, urban overcrowding, and exposure to crime can significantly affect individuals differently, particularly in terms of stress and anxiety levels. Vulnerable populations may be more sensitive to these environmental stressors (10).
- Sunlight and skin sensitivity, where individuals with certain skin types or genetic conditions (such as xeroderma pigmentosum) are particularly sensitive to ultraviolet (UV) radiation, increasing their risk of skin damage and cancers (11).
- Temperature sensitivity, where some individuals have a heightened sensitivity to temperature changes, can be particularly notable in conditions like fibromyalgia, where environmental temperature can exacerbate symptoms (12).

One of the known individual sensitivities affecting human health is radiation sensitivity:

- Where certain individuals, due to genetic factors or pre-existing health conditions, may have a higher risk of developing radiation-induced health issues, such as cancer (13).

It is known that even a slightly elevated level of environmental sensitivity may lead to worse ratings of the environment with no clear relation to the real environment (14). Consequently, environmental sensitivity should be considered as a confounding factor in environmental exposure studies. The independence from real exposure levels is in line with the results from studies showing that the differences in environmental ratings are also driven by psychological factors (14). Hence, both psychology and physiology research methods need to be used when studying individual sensitivities.

## Radiation sensitivity

Individual sensitivity to radiation is a well-known and scientifically established phenomenon. Because of the genetic and epigenetic differences between people, different persons may respond physiologically in different ways to exposure to the same radiation exposure. The phenomenon of individual sensitivity to radiation has been described for ionizing radiation (15–17) and for non-ionizing radiation, e.g., ultraviolet radiation (18, 19) or ultrasound (20).

The recently published opinion/review (21) has concluded that while it is well known that different persons can react differently to the same ionizing radiation exposure, the validated biomarkers of either tissue or stochastic effects have not been identified to date.

Search for biomarkers of ionizing radiation (hyper)-sensitivity is ongoing because about 5–10% of radiotherapy patients show severe damage sensitivity in healthy tissue irradiated collaterally during irradiation of pathological tissue (22). The nature and prediction of ionizing radiation hypersensitivity is not possible due to a lack of research that would identify specific biomarkers. The pre-therapeutic identification of radiosensitive patients would allow improvement of individual patients' treatment because hypersensitive patients could be excluded from high-dose radiation treatments and different therapies could be proposed whereas non-sensitive patients could profit even more from enhanced radiation therapy (23–25). Several studies were performed to find biomarkers of ionizing radiation hypersensitivity. However, current studies are difficult to compare with each other due to a lack of standardization of methods and high inter-laboratory variations. Despite these technical problems, the results of these studies support the notion that it will be possible to find the biomarkers and improve patient therapy, and the search for biomarkers of hypersensitivity to ionizing radiation should proceed using high-throughput screening techniques (23).

## Individual sensitivity to wireless radiation

One of the recent additions to the list of environmental pollutants is electromagnetic radiation emitted by wireless communication devices and networks (wireless radiation). Wireless radiation can induce various biological effects in cells grown *in vitro*, in experimental animals, and in humans. The reasons why the epidemic of such health problems like e.g., brain cancer did not materialize as predicted by some scientists might be that wireless-radiation-induced biological effects might affect health only in some particularly sensitive individuals, a minority of the total population. Also, changes in technology, such like the relocation of the mobile phone antenna from the top to the bottom of the phone set, changed how the brain tissue is irradiated.

Research into individual sensitivity to wireless radiation has garnered significant attention as the prevalence of mobile devices and wireless technology increases. Individuals report experiencing various symptoms they attribute to wireless radiation exposure, a phenomenon often referred to as electromagnetic hypersensitivity (EHS). Electromagnetic hypersensitivity is characterized by a collection of symptoms that individuals attribute to exposure to wireless radiation, including headaches, fatigue, stress, sleep disturbances, and skin symptoms. However, a clear scientific consensus on the existence and mechanisms of EHS remains elusive. Studies indicate variability in prevalence rates of self-reported EHS ranging from 1 to 10%. This diversity can partly be attributed to differing levels of public awareness and reporting.

Scientific research on EHS consists of three types of studies: (i) survey studies, (ii) provocation studies, and (iii) biochemical and physiological studies. The three types of studies have major overarching drawbacks (26). Firstly, researchers do not know whether the self-declared EHS persons volunteering in research projects have EHS because there are no diagnostic criteria for determining it. The group of self-declared EHS persons participating in the research study might be contaminated by the misdiagnosed EHS persons. In extreme situations, none of the self-declared EHS volunteers suffers from EHS. Secondly, scientists analyze solely the effects of exposures to wireless radiation and do not address, simultaneously occurring in real life, co-exposures to other environmental pollutants, like chemicals, particulate matter, or radiations other than wireless, which might have synergistic effects. Thirdly, all the experimental data obtained with the help of self-declared EHS volunteers is subjective and prone to bias caused by the beliefs and opinions of the volunteers (26). Conclusions of the provocation studies performed using psychology methods might be affected or even invalidated because of the existence of the placebo and nocebo phenomena. Placebo and nocebo indicate the ability of the human mind to affect the physiology of the human body. There is a well-known phenomenon among medical students of the “medical students' disease”. It is a condition frequently reported in medical students, who perceive themselves to experience symptoms of a disease they are studying. This condition is associated with the fear of contracting the disease in question. The same is likely happening when researchers show the study subjects' films presenting the dangers of wireless radiation exposure. It is obvious and expected that some persons will afterward “experience” some of the symptoms presented in the film. Furthermore, all volunteers have preconceived opinions on EMF and health. Thus, claims that news media reports and dissemination of information about precautionary measures to avoid exposure to wireless radiation cause a rise in the occurrence of EHS is incorrect and was shown to be so (27). The responses of the self-diagnosed EHS persons given during the provocation experiments are influenced by their pre-existing opinions about EHS. The data collected in the psychological provocation studies is not only subjective but is affected to an unknown degree by pre-existing opinions.

Search for sensitive individuals, most commonly using provocation studies where experimentally controlled exposures are followed by inquiries about acutely occurring symptoms and feelings, has failed to detect any sensitivity to wireless radiation. The reason might be that provocation exposures combined with psychological inquiries might be not enough sensitive to detect individual sensitivity to a single agent present in a mix of other environmental agents (26).

Some of the problems with provocation studies are:

- Capturing general sensitivities but not testing for them in the crossover study,
- Use of a generalized test approach that expects all EHS to respond in the same way,
- Testing EHS people with frequencies they report they are not sensitive to,
- Not always including objective biological tests,
- Insufficient recovery time between exposures,
- No accounting for latent effects (EHS people are not like light switches),
- Not tracking symptoms progression or remission over time,
- Pooling results what may hide sensitive individuals in group of non-sensitives—what calls for more personalized tests examining each person individually.

The practical problems of research on individual sensitivity and biomarkers of response to ionizing radiation (21) very closely resemble the problems of the research on non-ionizing radiation emitted by wireless communication devices (26, 28).

The scientific evidence surrounding individual sensitivity to EMF is complex and often inconclusive. While some studies suggest that reported symptoms in EHS individuals may not correlate with objective measures of wireless radiation exposure, the psychological and psychosomatic factors may play significant roles. Continued research is essential to deepen our understanding of EHS, its symptoms, and the underlying mechanisms at play (26).

Due to inter-individual biochemical differences, physiological and biochemical experiments on human volunteers will likely be necessary to determine whether some individuals react differently to wireless exposures.

## Biochemical individuality

Another scientific concept that supports the notion that individual sensitivity to wireless radiation needs to be studied using biochemical methods is the biochemical individuality concept. Biochemical individuality is the concept that each person has a unique biochemistry that influences how they respond to nutrients, drugs, and other factors. It suggests that individuals may have differing nutritional requirements, metabolic processes, differing responses to medications, and environmental factors based on their genetic makeup and environmental influences. This idea highlights the importance of personalized approaches to health and wellness, taking into account the individual differences in biochemistry among people. The concept of biochemical individuality was established by Roger J. Williams and it states that there is no such thing as an average person (29). We are all genetically and biologically unique. When sperm fertilizes the egg, our characteristics are not locked in stone. The concept of biochemical individuality argues that genes do not necessarily cause disease by themselves, but that nutrition and environmental factors can alter the outcome. The concept of biochemical individuality explains why some of us are better at detoxifying drugs and chemicals, why cancer genes respond in different ways to diet and environment, why some people are alcoholics or diabetics, why low-fat diets cause some people to gain weight, or why one person needs higher levels of a nutrient than another to stay healthy.

## A new research approach is needed to study individual sensitivity to wireless radiation

The to-date proposed biomarkers of EHS are not known/proven to be affected by RF-EMF exposures. Therefore, use of these “biomarkers” identifies persons with some health problem but it is not known what causes it. Attempts of EHS diagnosis using current physiological and biochemical tests are inadequate. In some research studies (30) biochemical tests analyzed biological endpoints thought to correlate with the symptoms experienced by self-diagnosed EHS persons: high-sensitivity C-reactive protein (hs-CRP), vitamin D2-D3, histamine, IgE, protein S100B, nitrotyrosine (NTT), heat shock protein 70 (HSP70), heat shock protein 27 (HSP27), anti-O-myelin autoantibodies, hydroxy-melatonin sulfate, 6-Ω-creatinine. In addition to the biochemical tests, the blood flow in the temporal lobes of the brain was examined with a non-invasive method of ultrasonic tomosphygmography.

The observed changes in expression of the examined biochemical endpoints did occur only in the minority of the self-declared EHS persons what is not surprising in a multifactorial ailment.

Only 40% of self-declared EHS persons had an increase in histamine level. None of the proposed biomarkers was prevalent in EHS persons: hs-CRP increased in 15% of EHS, vitamin D2–D3 declined in 23.2% of EHS, histamine increased in 40% of EHS, IgE increased in 22% of EHS, protein S100B increased in 15.5% of EHS, nitro-tyrosine (NTT) increased in 29% of EHS, Hsp27, Hsp70 detected in 7–19% of EHS, antibody to O-myelin detected in 17–29% of EHS, melatonin to creatinine ratio declined in EHS but the variation was too large to provide a specific number for the ratio.

Brain blood flow, examined with ultrasonic tomosphygmography, was claimed to decline in 50.5% of self-declared EHS persons, but the actual results of the tests were never shown.

Most importantly, there is no evidence that any of the proposed biomarkers was affected by wireless radiation exposure *in vitro* or in humans. Only two of the examined and proposed biomarkers, Hsp70 and Hsp27, are known to be affected by wireless exposures in cells grown in the laboratory. However, it is not known if the same occurs in living humans. Hence, the diagnostic value of Hsp70 and Hsp27 remains unknown.

Lastly, there are no blinded studies examining a group of volunteers, consisting of sensitive and non-sensitive persons, where the scientists would pinpoint sensitive persons after examining the proposed biomarkers.

The same problem is with the numerous biomarkers and diagnostic criteria for EHS proposed by the European Academy for Environmental Medicine (EUROPAEM). The proposed biomarkers are based on the symptoms claimed by the self-declared EHS persons but they lack proof that the symptoms were caused by wireless radiation exposures and that the wireless radiation exposures can cause changes in the expression of the proposed biomarkers (31).

There is a need for human volunteer studies where the already proposed, and other potentially useful biomarkers, would be examined in groups of sensitive and non-sensitive persons, ethically exposed to wireless radiation.

This newly designed research should be a combination of human provocation studies and examination of molecular-level responses in wireless-radiation-exposed people. Because it is not possible to reliably identify sensitive persons, the group of volunteers should consist of self-declared sensitive and self-declared non-sensitive persons. The self-declarations of volunteers in the group should be blinded from the researchers until the full data is analyzed. Such research should look for corroborating evidence that individual sensitivity to wireless radiation exists and look for the molecular targets of exposures that are proven to be affected by wireless radiation exposures. Regrettably, the to-date performed biomarker studies relied solely on anecdotal evidence from self-diagnosed EHS persons and did not determine whether the biomarkers are indeed in any way affected by the RF-EMF exposures (30).

The way forward in EHS research is to discover biomarkers of EHS, molecules that are affected by wireless radiation exposure, by research using high-throughput screening techniques of proteomics, transcriptomics, and metabolomics (32, 33). For the start, proteomics might be the most promising of these methods.

## Proteomics

Proteomics investigates the interactions, function, composition, and structures of proteins and their cellular activities. Proteomics provides a better understanding of the structure, function, and physiology of the organism than genomics or transcriptomics. Changes in gene expression do not affect the physiology of the organism for as long as the gene expression changes are not translated into changes in the expression and activity of proteins. The level of transcription of a gene gives only a rough estimate of its level of expression into a protein because of the physiological regulation of mRNA production, translation, and degradation. Therefore, proteomics provides a much more robust and representative picture of the functioning cell than other forms of large-scale biology, such as the sequencing of genomes or the global analysis of gene expression. At present, strategies for proteomics research can be divided into discovery proteomics and targeted proteomics. Discovery proteomics is more concerned with protein screening and dynamics, while targeted proteomics focuses more on detecting target proteins/peptides to achieve absolute quantification. In the biomedical field, proteomics finds widespread application in cancer research and diagnosis, stem cell studies, and the diagnosis and research of infectious and non-infectious diseases. In addition, it plays a pivotal role in drug discovery and the emerging frontier of personalized medicine. The limitations of proteomics include complexity in analysis, lack of standardization in sample processing, risk of high false positivity, and dynamic range of sample limits the estimation of low abundance of proteins, or failure in the validation of biomarkers in a larger number of patients due to lack of antibodies.

Despite the mentioned limitations, proteomics is being increasingly used for identifying and validating biomarkers for various diseases, like e.g., cancer, cardiovascular diseases, or neurodegenerative disorders. Modern proteomics, and other high-throughput screening techniques, allow global evaluation of the changes in the cellular proteome, metabolome, transcriptome, and genome that will reveal biological effects of wireless radiation exposures that are impossible to predict based on the currently available knowledge. High-throughput screening techniques are already widely used in clinical research in the search for biomarkers of diseases (34–39) and environmental toxicology (40–43).

The search and identification of biomarkers play an essential role in disease diagnosis, prognosis, and the development of personalized medicine. Biomarkers in proteomics refer to measurable substances, typically proteins, which indicate a biological state or condition of an organism/organ and can be used for disease diagnosis, prognosis, or therapeutic monitoring. The high-throughput scale of proteomics allows researchers to rapidly identify and quantify a large variety of potential protein biomarkers, present in various biological samples. Hence, proteomics has become a vital tool in the search for biomarkers—measurable indicators of specific biological states or conditions.

Applications of proteomics in biomarker discovery research:

- Understanding of the mechanisms of diseases: proteomics allows for the identification of protein alterations that occur in diseases. These alterations can serve as potential biomarkers for early diagnosis and understanding of disease progression (44–48).
- Identification of diagnostic biomarkers: comparing the proteomes of healthy individuals with those of patients, permits identification of specific proteins that are differentially expressed (49–59).
- Biomarkers for therapeutic applications: proteomics can identify biomarkers that predict response to therapies, particularly in cancer treatment, enabling personalized medicine (60, 61).
- Biomarkers for prognostic applications: proteomic profiles can serve as prognostic markers to inform of the future potential disease outcomes and patient survival (62).
- Integration of protein biomarker discovery with genomics and metabolomics approaches: combining proteomics with genomics and metabolomics can enhance biomarker discovery and provide a more comprehensive understanding of disease (63).

In summary, proteomics is paving the way for the identification and validation of biomarkers across a spectrum of diseases. Proteomics is invaluable in identifying and validating biomarkers, contributing to advancements in diagnosis, prognosis, and treatment personalization. It is astounding that the research on biological and health effects of the exposures to man-made wireless radiation does not use more efficiently proteomics, and other high-throughput omics technologies, to discover the potential biological and health effects. As technology evolves, the integration of proteomic data with other omics will further enhance the biomarker discovery process, potentially leading to better patient outcomes.

The reasons why proteomics is not used to study the physiological effects of wireless radiation exposures in humans are difficult to understand and comprehend. Despite the advantages of research using proteomics methodology, over the last 20 years, only a few proteomics studies have examined proteome changes in response to wireless radiation exposures. Of these studies, only two were performed on human volunteers (Table 1). Other proteomics studies were performed using animal models and cell lines grown in the laboratory. A review of these studies shows that the knowledge about the physiological impact of long and short-term exposures to wireless radiation is very scarce. The knowledge about the effects of wireless radiation exposures on the physiological processes within the human body is close to non-existent. Proteomics data suggests possible effects but the evidence is too limited for any practical use. This lack of knowledge about the physiological impact of wireless radiation exposure on the human body, at a time when the vast majority of people use mobile phones and are exposed to wireless radiation, is astonishing.

**Table 1:** List of studies that examined effects of exposures to wireless radiation on proteome.

**Type of study**	**Biological model**	**Result**	**Reference**
*In vivo*	Mice	Induction of emotional behavior alteration	(64)
*In vivo*	Mice	Broad protein expression changes in the brain	(65)
*In vitro*	Mice	Ca. 1% of proteome responds by small changes in protein abundance	(66)
*In vivo*	Rats	Deregulation of hippocampus proteome	(67)
*In vivo*	Rats	Increases in testicular proteins, carcinogenic risk, and reproductive damage	(68)
*In vivo*	Rats	A 30-day exposure induces non-thermal stress in testicular tissue	(69)
*In vivo*	Human	Altered protein profile of chorionic tissue of early pregnancy	(70)
*In vivo*	Human	Change in protein expression profile in the skin	(71)
*In vitro*	Human	Ca. 1% of proteome responds by small changes in protein abundance; considered insignificant effect	(66)
*In vitro*	Human	Differential expression in 8 proteins	(72)
*In vitro*	Human	Lack of statistically significant changes in protein expression	(73)
*In vitro*	Human	An 8-h exposure caused a significant increase in protein synthesis	(74)
*In vitro*	Human	900 and 1,800 GSM affect the expression of some proteins	(75)
*In vitro*	Human	Changes in 4 proteins were detected and re-confirmed as upregulated	(76)
*In vitro*	Human	No convincing evidence of an effect on protein expression	(77)
*In vitro*	Human	Cell responses might be genome- and proteome-dependent	(78)
*In vitro*	Human	Several proteins with altered expression levels, e.g., cytoskeleton	(79)
*In vitro*	Human	Changes the phosphorylation and expression of numerous proteins	(80)
*In vitro*	Human	Changes in phosphorylation and expression of numerous proteins	(81)

There are numerous advantages of using proteomics in the search for biomarkers of individual sensitivity to wireless radiation (82–84):

- Comprehensiveness of analysis—proteomics allows for the simultaneous analysis of thousands of proteins in a sample. This comprehensive approach can help identify specific proteins involved in the cellular response to radiation, which might not be captured by other techniques like genomics or transcriptomics.
- Providing functional insights—proteins are the functional molecules in the cell, and their levels and modifications can provide direct information about biological processes, pathways, and mechanisms. This functional insight is critical for understanding how individuals might respond differently to radiation exposure.
- Analysis of the dynamic range of response—the proteome can change rapidly in response to stimuli, such as radiation exposure. Proteomics can capture these dynamic changes in protein expression and post-translational modifications more effectively than methods that assess static genetic information.
- Integration of proteomics with other omics techniques, like genomics and metabolomics, will give a more holistic view of the biological response to radiation. This multi-omics approach can elucidate complex interactions and pathways relevant to individual sensitivity.
- Identifying biomarkers in wireless radiation exposed persons—through proteomic studies, researchers can identify potential biomarkers that correlate with sensitivity to radiation. This can facilitate the identification of sensitive individuals, and personalized medicine approaches, and help in predicting treatment responses.

## Summary conclusion

In conclusion, it is logical to conclude that the individual sensitivity to wireless radiation emitted by wireless communication devices and networks exists and impacts the health of sensitive persons. Clearly, the to-date unsuccessfully used methods of provocation studies were either too crude or too much affected by the perceptions and preexisting opinions of study volunteers. A combination of psychological inquiry and physiological/biochemical examination of the responses to wireless radiation exposures are necessary to detect and define diagnostic criteria of individual sensitivity to wireless radiation. In physiological/biochemical testing, while no single method can fully capture the complexities of biological responses to radiation, proteomics offers unique insights that are crucial for understanding individual sensitivity. It should be viewed as an integral part of a broader set of tools used to study radiation effects. Proteomics and other high-throughput “omics” screening techniques should and must be broadly introduced to wireless radiation bio-effects and bio-markers research.
